# Perspective taking as a mechanism for children’s developing preferences for equitable distributions

**DOI:** 10.1038/s41598-021-84968-2

**Published:** 2021-03-11

**Authors:** David M. Sobel, Jayd Blankenship

**Affiliations:** Department of Cognitive, Linguistic, and Psychological Sciences, Brown University, 190 Thayer St., Box 1821, Providence, RI 02912 USA

**Keywords:** Psychology, Human behaviour

## Abstract

How do young children develop a concept of equity? Infants prefer dividing resources equally and expect others to make such distributions. Between the ages of 3–8, children begin to exhibit preferences to avoid inequitable outcomes in their distributions, dividing resources unequally if the result of that distribution is a more equitable outcome. Four studies investigated children’s developing preferences for generating equitable distributions, focusing on the mechanisms for this development. Children were presented with two characters with different amount of resources, and then a third character who will distribute more resources to them. Three- to 8-year-olds were asked whether the third character should give an equal number of resources to the recipients, preserving the inequity, or an unequal number to them, creating an equitable outcome. Starting at age 7, children showed a preference for equitable distributions (Study 1, N = 144). Studies 2a (N = 72) and 2b (N = 48) suggest that this development is independent of children’s numerical competence. When asked to take the perspective of the recipient with fewer resources, 3- to 6-year-olds were more likely to make an equitable distribution (Study 3, N = 122). These data suggest that social perspective taking underlies children’s prosocial actions, and supports the hypothesis that their spontaneous capacity to take others’ perspectives develops during the early elementary-school years.

## Introduction

Children undergo developmental changes in their preferences for allocating resources^1,2^. Infants between the ages of 15–21 months prefer to make equal distributions (i.e., giving recipients the same amount of resources) and expect others to distribute resources in an equal manner^3–5^. Three- and 4-year-olds articulate dividing resources equally as a social norm of fairness, and generate equal distributions among third-party others. However, they often endorse unfair distributions when they are the beneficiary of the inequity^6^. It is not until after the preschool years (usually ages 7–8) that children show preferences for avoiding inequities when they are the beneficiary of the inequity^7,8^, and this appears to differ among cultures^9^.

Generating *equal* distributions, in which recipients are given the same amount of resources is not necessarily the same as generating *equitable* distributions, in which recipients end with or move towards having the same amount of resources, or in which recipients receive resources commensurate with situational factors leading up to the distribution. An example is effort. An equal distribution is expected when two recipients collaborate equally; but if there is an unequal amount of work required to achieve a reward, a more equitable distribution might be to give more resources to the individual who worked harder. Preschoolers engage in such meritorious reasoning; they do not provide equal distributions to recipients who demonstrate different effort^7,10,11^. Similarly, preschoolers integrate the value of objects when allocating resources. Three- to 6-year-olds create equal distributions when asked to give resources to others (in terms of number of objects), but give away the objects they value less to less preferred others^12,13^. These findings suggest that young children integrate knowledge of social norms into judgments of equity by distributing resources or accepting distributions in ways that are not necessarily just a 1–1 correspondence between resource and recipient.

Another example that has been studied concerns the relation between resources that recipients receive through windfall gains (i.e., without putting in effort) and the existing resources possessed by recipients. If two recipients start with a different amount of resources, distributions of windfall gains can be equal to preserve the overall inequity, or distributions can be unequal to attempt to rectify the inequity. In one example^14^, 3- to 8-year-olds were presented with stories about two characters who come from different places with different histories of wealth; one character had a lot of resources and the other had none. Children were then given six resources and they examined how children physically divided those resources among the two recipients. Three- and 4-year-olds exhibited preferences for equality, but gave more to the recipient with fewer resources between ages 5–8. Other investigations on rectifying inequities show similar developmental pattern^8,15–18^.

In many of these cases, children are given a set of resources to distribute. In doing so, children might create an equal distribution by giving the same number of resources to each recipient or an equitable outcome by giving a different number of resources to each recipient such that they have the same number (or closer to the same number) after the distribution. However, children might also generate other kinds of distributions that fit neither pattern. To examine the developmental trajectory of children’s preferences for equal or equitable actions, we used a simple choice method in which children were asked to choose which of two distributions they endorsed. This way, children had to explicitly choose an end state. This also addressed a concern with the choice method, as it is possible that asking children to distribute resources might have them deal resources in a 1-1 manner simply because they’ve seen others act in this way. This would result in an equal distribution without children necessarily understanding equality, or result in an unequal distribution if children err (e.g., accidentally deal 2 resources to someone on a turn). Having children choose the outcome potentially reduces this demand characteristic.

Our goal was to examine whether children would choose equal distributions (giving the same number of resources to each recipient) or whether children would choose an equitable distribution (such that the recipients have the same number of resources after the distribution). Similar to previous studies, we expect children’s preference for equity over equality to emerge between the ages of 5–8, but we also tested 3–4-year-olds to consider whether our measure affected preschoolers’ performance. After establishing this basic finding (Study 1), we consider two potential mechanisms for this developmental trajectory (Studies 2a, 2b, and 3).

The first mechanism we considered is numerical competence. To recognize an inequity, children must understand that individuals have greater or fewer resources. The simplest way this could be done is through understanding ordinal relations (that is, that one person has more or less than another), which is understood by 15-month-olds in looking time measures^19^, but might involve more explicit comparisons of numericities, which develops between ages 3–5^20,21^. When asked to distribute resources between two recipients, only preschoolers who understood the cardinal principles (the connection between a number word and its exact quantity) could remember the distributions they generated, and avoided using simple heuristics to come to an equal distribution^22,23^. These studies, however, centered on generating a distribution to recipients who started with no resources; here we consider distributing resources to recipients who start with an unequal amount to see whether registering that the recipients initially have different amounts of resources relates to children’s preference for equitable distributions (Studies 2a and 2b).

We then consider a second mechanism in Study 3, which is that children’s emerging preference for equity is influenced by social perspective taking. Asking 3- to 6-year-olds to consider how recipients or how they themselves would react to an inequity led to higher levels of generosity in a mini-dictator game^24^. Similarly, 6- to 9-year-olds showed relations between their performance on a measure of others’ emotional understanding and their advantageous inequity aversion in an inequity game^25^. Three- to 5-year-olds who were primed to take the emotional perspective of another person who experienced a negative event, were more likely to ignore an experimenter’s request to create an inequity between two recipients or even between themselves and a third-person when the inequity benefitted them; children trained to take the conceptual perspective of another person who experienced a surprising event were more likely to comply with the experimenter^26^. These studies all suggest that perspective taking, and theory of mind capacities more generally, play an important role of children’s developing understanding of fairness^27^. Given these findings, we predicted that preferences for equity over equality might emerge when children were asked to take the perspective of the recipient with fewer resources.

**Figure 1:**
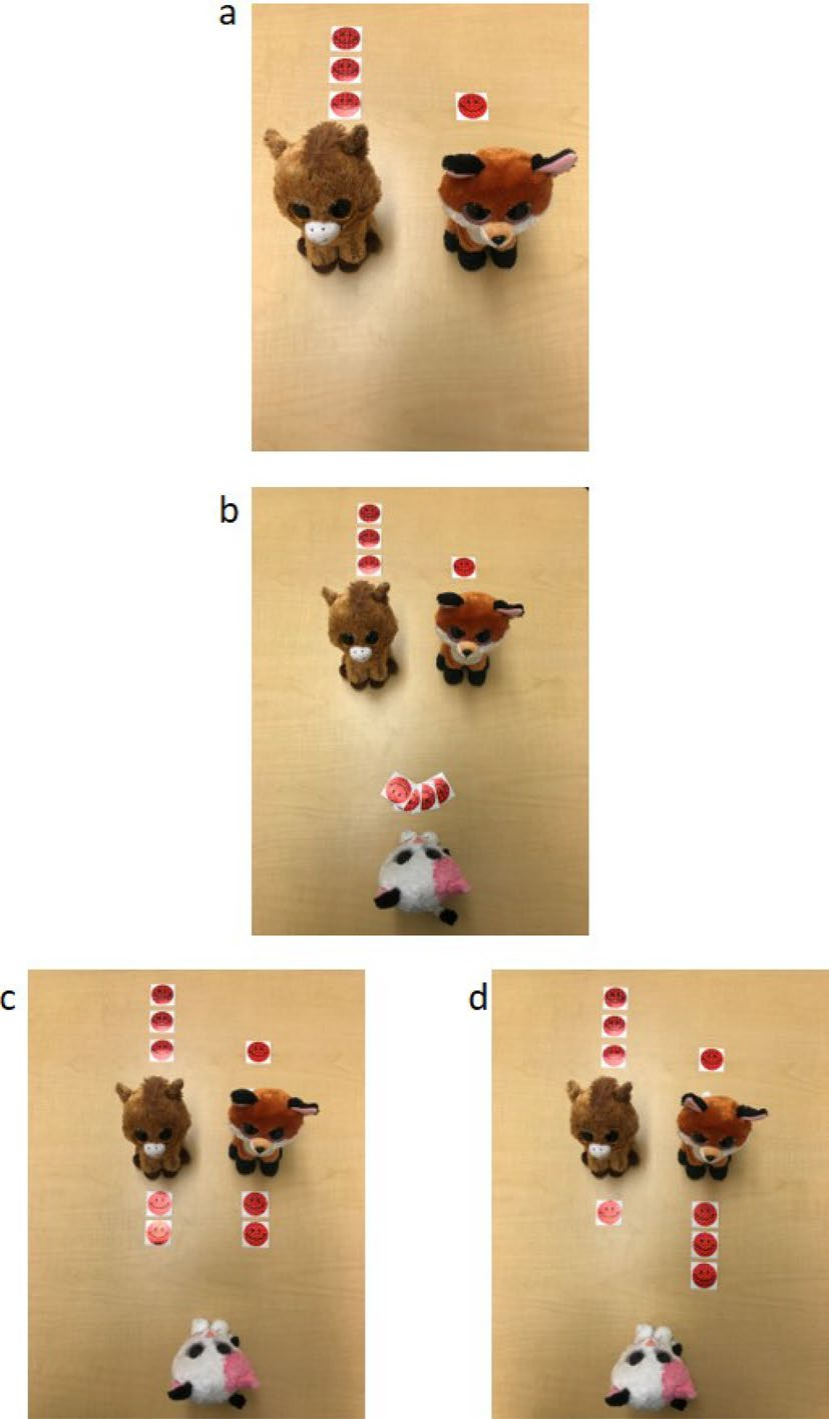
(**a**–**d**) Pictures used throughout the studies. Children were introduced to two characters (Pony and Foxy) with different resources (**a**), then a character (Kitty) who would give more resources to Pony and Foxy (**b**). Children were asked which distribution Kitty should make – one where both recipients get the same number, but where the inequity is preserved (**c**) or one where the recipients get different numbers, but where the final outcome is equitably (**d**). See methods for details.

In Study 1, 3- to 8-year-olds were introduced to two characters, Pony and Foxy. One character had 3 stickers (a resource that was pretested to be attractive to children at these ages); the other character had 1 sticker (see Fig. 1a). No other information about the characters was presented to ensure children only used the inequity in the status quo as the basis of their response. A third character (Kitty) came along and had four new stickers (Fig. 1b). Kitty stated that she was going to give these stickers to Pony and Foxy. Children were asked what Kitty should do while being presented with two pictures detailing the possibilities and were asked to choose between two options. In the first (Fig. 1c), Kitty gave two stickers to each recipient—an equal distribution but an inequitable outcome. In the second (Fig. 1d), Kitty gave one sticker to the recipient with three and three stickers to the recipient with one—an unequal distribution but an equitable outcome. In Study 2a, a new group of 3- to 8-year-olds received a similar procedure, but one that measured whether they recognized there was a final inequity between Pony and Foxy. In Study 2b, children received both the procedures in Study 1 and 2a. Finally, in Study 3, children received a similar version of the procedure from Study 1, but one where they were asked to take the perspective of one of the recipients.

## Results

All data files for these studies are available at https://osf.io/mbfpj. Overall, in Study 1, children chose the equitable over the equal outcome 60% of the time, significantly more often than chance, Binomial test, *p* = 0.02. We performed a binary logistic regression on responses with age (in months) and gender as independent measures. We included gender in our models because some researchers have suggested that males and females respond differently in prosocial behavioral tasks, particularly when those measures have affective perspective-taking components^28^. The overall model was significant compared to an intercept-only model, χ^2^(2) = 17.81, *p* < .001. Age was a significant factor, B = 0.04, SE = 0.01, Wald χ^2^(1) = 15.67, *p* < .001, OR = 1.04, 95% CI [1.02, 1.06]. Gender was not significant, B = 0.12, SE = 0.36, Wald χ^2^(1) = 0.11, *p* = .74.

**Figure 2:**
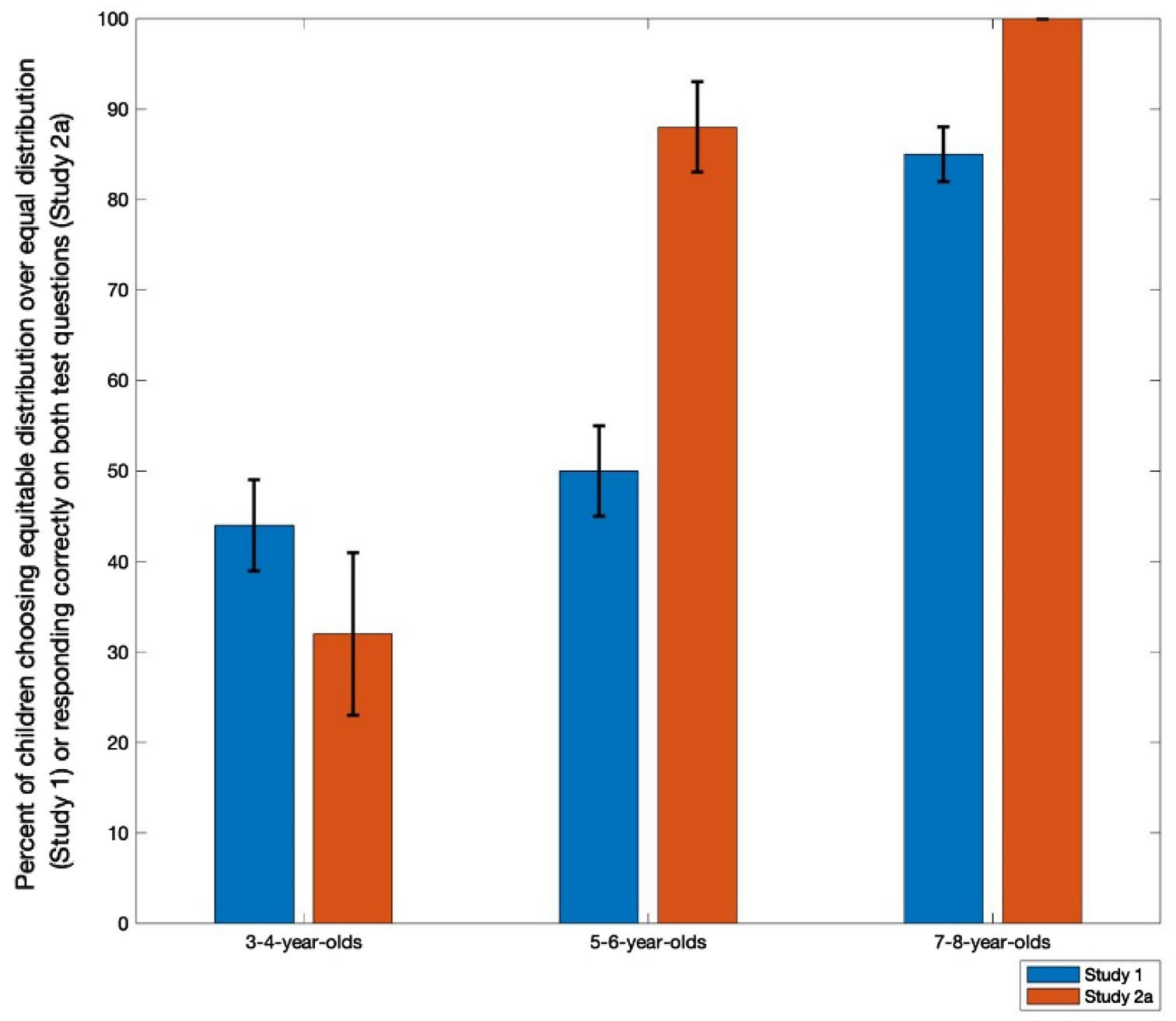
Percentage of children who chose the equitable distribution in Study 1 and responded correctly to the two number questions in Study 2a.

To analyze the role of age further, we divided children into three equal age groups (3–4-year-olds, 5–6-year-olds, and 7–8-year-olds). Their responses are shown in Fig. 2 (Top Panel). Only the oldest age group chose the equitable outcome differently from chance, Binomial tests, *p* = .43 for the 3–4-year-olds; *p* = 1.00 for the 5–6-year-olds; *p* < .001 for the 7–8-year-olds. These findings are consistent with the previous research establishing preferences for equity over equality by age 7.

Having established this paradigm as a method for testing children’s preferences for equality vs. equity, we examine what potentially underlies this development. In Study 2a, we considered whether 3- to 8-year-olds understood that the outcomes of the distributions would result in equal or unequal distributions of resources, and whether that knowledge affected the choices younger children made in their distributions. Children were shown the two distribution pictures from Study 1 (Fig. 1c,d), and were asked whether one recipient had more stickers or if they had the same amount.

The proportion of children who responded correctly to both of these questions is shown in Fig. 2. Children responded correctly on both questions 74% of the time, significantly more frequently than chance, Binomial test, *p* < .001. We performed a binary logistic regression on correct responses on both questions with age (in months) and gender as independent measures. The overall model was significant compared to an intercept-only model, χ^2^(2) = 35.02, *p* < .001. Age was a significant factor, B = 0.12, SE = 0.03, Wald χ^2^(1) = 14.72, *p* < .001, OR = 1.13, 95%CI [1.06, 1.21]. Gender was not significant, B = -0.26, SE = 0.72, Wald χ^2^(1) = 0.68, *p* = .74. We broke responses into the same three age groups as in Study 1. Three- and 4-year-olds (33%) responded no differently from chance, Binomial test, *p* = .23, while 5- and 6-year-olds (87%) and 7- and 8-year-olds (100%) were both above chance levels, Binomial tests, both *p *values < .001. These data suggest that children responded correctly on the questions in Study 2a at earlier ages than their preference for equitable distributions in Study 1. They also suggest that the youngest age group in Study 1 might not have understood that the distributions they could create could result in an inequity.

**Figure 3:**
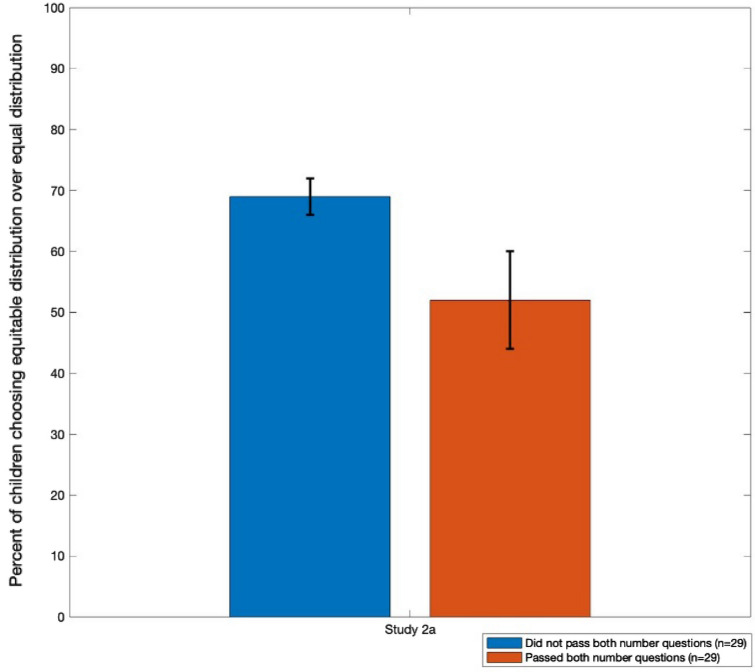
Percentage of children who chose the equitable distribution in Study 2b depending on whether they respond correctly to the two number questions (second picture).

Study 2b examined the possibility that these youngest children would choose to make equitable distributions if they understood that one outcome resulted in the recipients having the same amount of resources and the other resulted in an inequity. We presented 3- to 5-year-olds with the procedures from Studies 1 and 2a (using different pictures). These data are shown in Fig. 3. Overall, children chose the equitable over the equal response 63% of the time, which was marginally different from chance responding, Binomial test, *p* = .055. Forty percent of the children responded correctly on both numerical question, which was significantly different from chance, Binomial test, *p* = .02.

We ran a binary logistic regression on whether children chose the equitable response, looking at age, gender, and their performance on the numerical questions as independent variables. The overall model was not significant compared to an intercept-only model, χ^2^(3) = 2.13, *p* = .55, and none of the three independent variables significantly predicted children’s responses, all Wald χ^2^(1)-values < 0.64, all *p* values > .69. These data suggest that the development of a preference for equitable distributions is not based simply on children’s developing knowledge of who has more or fewer resources after the distribution. Five-year-olds can register who has more or fewer resources in the outcome pictures, but Study 1 showed that is not until children are 7 that they show a preference for equitable distributions. Understanding distributions will result in equitable outcomes is potentially necessary for generating such distributions, but not sufficient.

We also considered the possibility that this development related to 7- and 8-year-olds being able to register the perspective of the recipients spontaneously. Numerous studies suggest the importance of perspective taking in preschoolers’ empathy^29^. If 3–6-year-olds (who did not show a preference for equity in Study 1) were asked to attend to the recipients individually, children might be more likely to take the perspective of each recipient in constructing a distribution. When asked to attend to the recipient who started with fewer resources, children might be more likely to register that equally distributing the resources (i.e., giving the recipients the same number) would preserve a negative outcome to that individual, thus leading them towards an equitable distribution (giving the recipients stickers so that they had the same number at the end).

**Figure 4:**
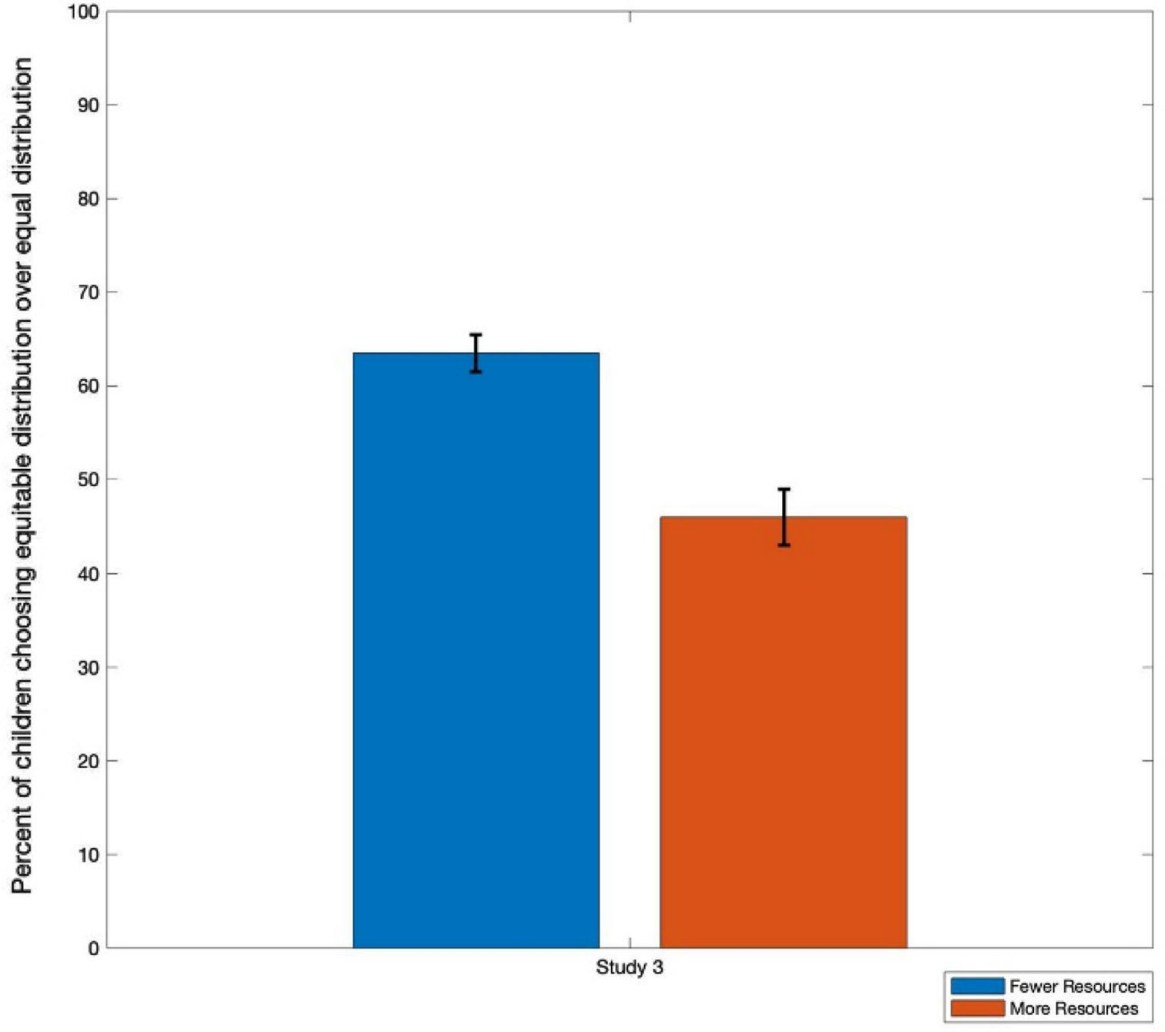
Percentage of children who chose the equitable distribution in Study 3 depending on which perspective children are asked to take.

Study 3 replicated the procedure of Study 1 on two groups of 3- to 6-year-olds. One group was asked to respond for the recipient who had more resources initially; the other was asked to respond for the recipient who had fewer resources initially. This required children to take the perspective of one of these recipients. Responses are shown in Fig. 4. Sixty-four percent of the children who were asked to respond for the recipient with one sticker chose the equitable distribution, while 46% of the children who were asked to respond for the recipient with three stickers did so. The distribution of responses between the conditions differed, χ^2^(1, N = 122) = 4.01, *p* = .05, Phi = .18.

We conducted a binary logistic regression on whether children generated an equitable distribution, with age, gender, and whether children were asked to respond as the recipient with 3 or 1 stickers as independent variables. The overall model was not significant, compared to an intercept-only model, χ^2^(3) = 5.82, *p* = .12. However, whether children responded for the recipient with 3 or 1 stickers was a significant factor in this model, B = 0.74, SE = 0.37, Wald χ^2^(1) = 3.96, *p* = .05, OR = 2.10, 95%CI [1.01, 4.37]. Age and gender were not significant predictors in the model, B = -0.02 and 0.17, SE = 0.02 and 0.38, Wald χ^2^(1) = 1.56 and 0.20, *p* = .21 and .65 respectively. Moreover, if one builds a model that includes the interactions among these variables, the overall model is not significant compared to an intercept-only model, nor is any main effect significant including perspective. However, if one builds a model with only perspective, that model is significantly different from an intercept-only model, χ^2^(1) = 4.03, *p* = .05 and perspective is a significant predictor (as shown above). The model with only perspective as an independent variable is also a better fit to the data, as measured by BIC (167.98 vs. 175.80).

## Discussion

We investigated the mechanisms behind children’s developing preferences for equitable distributions – in this case, where distributions are made by considering the amount of resources recipients initially have. Starting at age 7, children in Study 1 showed a clear preference for rectifying the initial inequity between the two recipients by unequally distributing resources such that both recipients had the same amount at the end. This finding is consistent with several findings that show children also make similar distributions^7,8^. Similarly, in an ultimatum game, 7–8-year-olds, 11–15-year-olds and adults all showed similar levels of inequity aversion when given the option of generating an equitably outcome^30^. In contrast, 5-year-olds appreciated that fair distributions in an ultimatum game were equal ones^31^.

Some studies have found that children’s numerical knowledge relates to their ability to create fair distributions^22^ or resolve inequities^23^. Study 2a suggests that children develop the capacity to appreciate that there is a final inequity in the distribution around age 5, before they reliably chose to rectify that inequity with their distribution (in Study 1). Study 2b suggests that for 3- to 5-year-olds (who might not reliably recognize the inequity), the ability to do so does not relate to their decision to rectify it. Children’s numerical knowledge might be necessary to appreciate resource inequities, but it does not appear sufficient to cause children to rectify those inequities.

In Study 3, when 3- to 6-year-olds (children who were not reliably rectifying the inequity in Study 1) were prompted to take the perspective of the recipient with fewer resources, they were slightly more inclined to endorse a distribution that resulted in the two recipients having the same number of resources. When children were asked to take the perspective of the recipient with the greater number of resources initially, they responded no differently from chance, nor differently from how they responded in Study 1, where they were asked to respond for the distributor.

This difference in response patterns between the conditions addresses an alternative interpretation of these data. Children might be responding on the basis of maximizing the number of stickers that the recipient receives, which would motivate their choice when asked to take the perspective of the recipient with only one sticker. That children do not do this when asked to take the perspective of the recipient who started with three stickers speaks against this interpretation. It is possible that children are faced with a dilemma in this condition; when asked to take the perspective of the recipient with more resources, children might believe that that recipient wants to maximize resources or might believe that that recipient wants to ensure equity (thus leading to the chance-level responding). However, we caution against such a strong claim, particularly given the fact that the effect size of perspective taking, while significant, was relatively small and might have been influenced by other factors (as indicated by the absence of a significant effect when interactions with age or gender were considered). This is consistent with other studies that have related theory of mind with children’s fairness, which also show similar significant, but weak effects^25^. Instead, we suggest that children who are asked to take the perspective of the recipient with fewer resources might be primed to equalize the outcome (or simply maximize their stickers), but being asked to take the perspective of the recipient with more resources at the outset does not necessarily affect how children respond. To further test this, future investigations should consider how children would divide resources among recipients with an existing inequity where the options are only an equal distribution or one that increases the inequity. An open question is whether children will attempt to move towards rectifying the inequity or maximize resources when asked to take the perspective of the beneficiary. It would also be interesting to further consider whether children are making exclusively third-person judgments or potentially maximizing resources for themselves.

To conclude, consistent with other investigations^7,8,30^, a preference for equity over equality in crafting distributions of resources emerged at age 7. This preference seemed less related to children’s numerical knowledge than to their perspective taking abilities. This suggests the possibility that by the age of 7, children might spontaneously register the inequity in possessed resources of the two recipients, and move to rectify that inequity. Spontaneous perspective-taking has often been demonstrated in adults’ inability to inhibit others’ visual perspective when reporting on their own^32^. Six- to 10-year-olds showed similar effects in a social, but not a non-social task^33^. We suggest that by this age children in this sample might be spontaneously taking the perspective of the recipient with fewer resources to resolve the inequity through their choice of distribution. While it is certainly possible that there are alternate interpretations of the present set of findings, and the use of alternative methods like eye tracking could provide inside into such interpretations, we think this hypothesis is a promising for further courses of study.

## Method

All studies were approved by the IRB of Brown University under protocol #1701001674, *Belief revision in early childhood: Learning about learning in the lab and museum*. All methods were carried out in accordance with relevant guidelines and regulations and informed consent was obtained for all participants from a parent or legal guardian. All children were recruited from a list of laboratory birth records and tested at Brown University in a laboratory or from the floor of Providence Children’s Museum and tested in a dedicated testing space at the museum. Parents were always present during children’s participation.

In Study 1, we tested 24 children in each year from ages 3 to 8 (N = 144, 76 girls, 68 boys, *M*_*age*_ = 71.23, Range = 36.00–107.70 months; SD = 20.58 months). This sample size was determined via power analysis for comparing ages (by year) assuming α = 0.05, β = 0.20 and a large effect size, w = 0.50. Children were shown the photo in Fig. 1a, and shown two stuffed animals (Pony and Fox). One had 3 red smiley-face stickers, the other had 1 (counterbalanced). Children were told the names of the animals and that each had stickers. Children then saw the photo in Fig. 1b, in which a third animal (Kitty) had 4 stickers, which she was going to give to Pony and Fox. Children were asked what Kitty should do while being presented with two pictures (Fig. 1c,d) detailing the possibilities (e.g., give Foxy two and give Pony two or give Foxy < who started with three for the sake of example > one and give Pony three).

In Study 2a, we presented a group of 3- to 8-year-olds (N = 72, 35 girls and 37 boys, *M*_*age*_ = 71.97 months, SD = 20.94 months, N chosen to ensure the same three age groups described in the results of Study 1 had n = 24 each) with the two test pictures from the previous study (shown in Fig. 1c,d). Children were introduced to the two stuffed animals in the same manner as Study 1 and told that the vertical row of stickers next to them were their stickers. Children were asked, “Who has more stickers? Does Pony have more, does Foxy have more, or are they the same?”

In Study 2b, we tested a group of 48 children between the ages of 3–5 (*M*_*age*_ = 53.62 months, SD = 10.64 months, 23 girls, 25 boys). In Study 2a, children at these ages responded at chance levels. A new set of pictures with three new animals and a different resource (a stuffed dog, elephant, and frog who had the same numbers of orange balloons) was constructed. Children were given the procedures used in Study 1 and Study 2a (counterbalanced for order). One procedure used the same stimuli from Study 1-2a; the other used the new set of stimuli (counterbalanced).

In Study 3, we tested a group of 122 children between the ages of 3–6 (*M*_*age*_ = 59.55 months, SD = 12.94 months, Range 36.3–83.40 months, 56 girls, 66 boys). Five additional children were tested, but not included in the analysis because of experimental error in administering the procedure. This age range was chosen because in Study 1, children in this age range responded at chance levels. The same procedure as in Study 1 was administered except the test question was different. When the third character was introduced (Kitty), Kitty said “I’m going to give these stickers to my friends, but I don’t know what to do.” Kitty then asks one of the two recipients what she should do. Half of the children were asked to respond for the recipient with three stickers while the other half were asked to respond for the recipient with one sticker (i.e., “Pony, what should I do?” and then the experimenter would ask the child, “What will Pony say…” while producing the pictures that showed the equal and equitable distributions).
